# Relationship between the height of fibular head and the incidence and severity of knee osteoarthritis

**DOI:** 10.1515/med-2022-0523

**Published:** 2022-07-22

**Authors:** Xinghui Xu, Jin Yang, Jun Li, Deping Yao, Pan Deng, Boliang Chen, Yifei Liu

**Affiliations:** Department of Joint Orthopaedic, Baoji Hospital of Traditional Chinese Medicine, Baoji, Shaanxi, 721000, China; Department of Traumatology, Orthopedics Hospital, Affiliated Hospital of Shaanxi University of Traditional Chinese Medicine, Shaanxi, China; Department of Radiology, Baoji Hospital of Traditional Chinese Medicine, Baoji, Shaanxi, 721000, China; Department of Joint Orthopaedic, The First Clinical Medical College, Guangzhou University of Chinese Medicine, Guangzhou, Guangdong 510405, China

**Keywords:** knee osteoarthritis, fibular head height, Kellgren–Lawrence grade, three-dimensional reconstruction, varus deformity

## Abstract

The purpose of this study was to investigate the correlation between fibular head height and the incidence and severity of osteoarthritis associated with varus knee deformity. The fibular head height, joint line convergence angle (JLCA) and medial proximal tibial angle (MPTA) were measured in a three-dimensional model. Ordinal multivariate logistic regression was used to analyze the correlation between fibular head height and Kellgren–Lawrence (K–L) grade. Pearson correlation was used to analyze the correlation between fibular head height and K–L grade. A total of 232 patients (232 knees) were finally included in the study. There were significant differences in JLCA and hip–knee–ankle angle (*P* < 0.05), and both JLCA and hip–knee–ankle angle increased with severe aggravation of K–L grade. Both fibular head height and MPTA decreased as the K–L grade was severely aggravated. There was a significant negative correlation between K–L grade and fibular head height (*r* = −0.812, *P* < 0.001). Furthermore, there was a significant negative correlation between fibular head height and hip–knee–ankle angle (*r* = −0.7905, *P* < 0.001). In addition to body mass index, fibular head height is a risk factor for the pathogenesis of osteoarthritis associated with varus knee deformity; the smaller the fibular head height, the more severe the degree of varus deformity.

## 1 Introduction

Knee osteoarthritis, a degenerative knee disease-based disease, is a common cause of knee pain and lower limb disability. In the United States, half of the population may develop knee osteoarthritis at the age of 85 years [1]. In addition, the study predicts that by 2030, the demand for total knee arthroplasty will be six times that of 2005 [2]. The medial compartment of the knee has an approximately 10-fold higher risk of morbidity than the lateral compartment [3] and is generally thought to be associated with higher long-term pressures in the medial compartment of the knee [4]. For degenerative changes in the knee joint, degenerative changes in the medial compartment of patients with osteoarthritis associated with varus knee deformity are generally assessed clinically by X-rays, and changes in the lateral structure are usually overlooked. While Zhang Yingze’s team found that there was an adaptive curve in the proximal fibula of patients with osteoarthritis associated with varus knee deformity [5], fibular weight bearing is one of the triggers of osteoarthritis associated with varus knee deformity, fibula bearing part of the body weight leads to greater force on the medial tibial plateau than on the lateral side and long-term high load leads to faster collapse of the medial tibial plateau than on the lateral side, that is, the “uneven subsidence” theory [6].

We have also found in the clinic that not only is there an adaptive curvature of the proximal fibula in patients with osteoarthritis associated with varus knee deformity, but the fibular head position is usually also high, even close to the level of the lateral tibial plateau. Our previous finite element studies found widening of the medial knee joint space and decreased medial compartment pressure after proximal fibular osteotomy (PFO) [7]. Patients significantly relieved symptoms such as knee pain and limited mobility after PFO surgery [8]. The fibular head height refers to the distance between the upper edge of the fibular head and the horizontal tangent passing through the lowest point of the lateral tibial plateau. Clinical practice and related studies have suggested that there may be an association between fibular head height and knee osteoarthritis. The change in fibular head height may be a manifestation of osteoarthritis or a risk factor for the occurrence and development of osteoarthritis. However, there is still a lack of relevant evidence-based evidence. X-rays are two-dimensional images and cannot assess the relative rotation between the tibia and fibula. In addition, the influence of knee rotation and flexion on the measurement results cannot be completely ruled out by using the knee X-ray film for measurement.

In this study, we measured fibular head height in a three-dimensional model of the knee joint. We hypothesized that fibular head height was associated with the onset and severity of osteoarthritis associated with varus knee deformity. The purpose of this study was to investigate whether fibular head height is an independent risk factor for the pathogenesis of osteoarthritis associated with varus knee deformity, to investigate the correlation between fibular head height and imaging findings grading of osteoarthritis associated with varus knee deformity and to investigate the correlation between fibular head height and the degree of varus deformity of the knee.

## 2 Materials and methods

### 2.1 General information

Knee patients who visited Baoji Hospital of Traditional Chinese Medicine from June 2018 to June 2020 were retrospectively analyzed. Inclusion criteria were as follows: (1) knee arthritis patients or healthy volunteers; (2) age 30–70 years; and (3) weight-bearing computed tomography (CT) and X-ray image data were complete. [A prototypic commercial CT scanner (LineUp, CurveBeam) was used to acquire weight-bearing images of knees.] Exclusion criteria were as follows: (1) previous history of lower limb trauma or surgery; (2) suffering from congenital knee deformity or skeletal dysplasia; (3) combined with diseases that seriously affect lower limb function, such as cerebral palsy, diabetic neuropathy and diabetic foot; and (4) combined with other knee joint diseases, such as knee joint tumors. All patients and their families gave informed consent to the treatment protocol, which was approved by the hospital ethics committee K.[2018] 038.


**Ethical approval:** This study was conducted in agreement with the Declaration of Helsinki and its later amendments or comparable ethical standards and had been approved by the ethics board of Baoji Hospital of Traditional Chinese Medicine (No: K.[2018]038).

### 2.2 Data measurement

**Figure 1:**
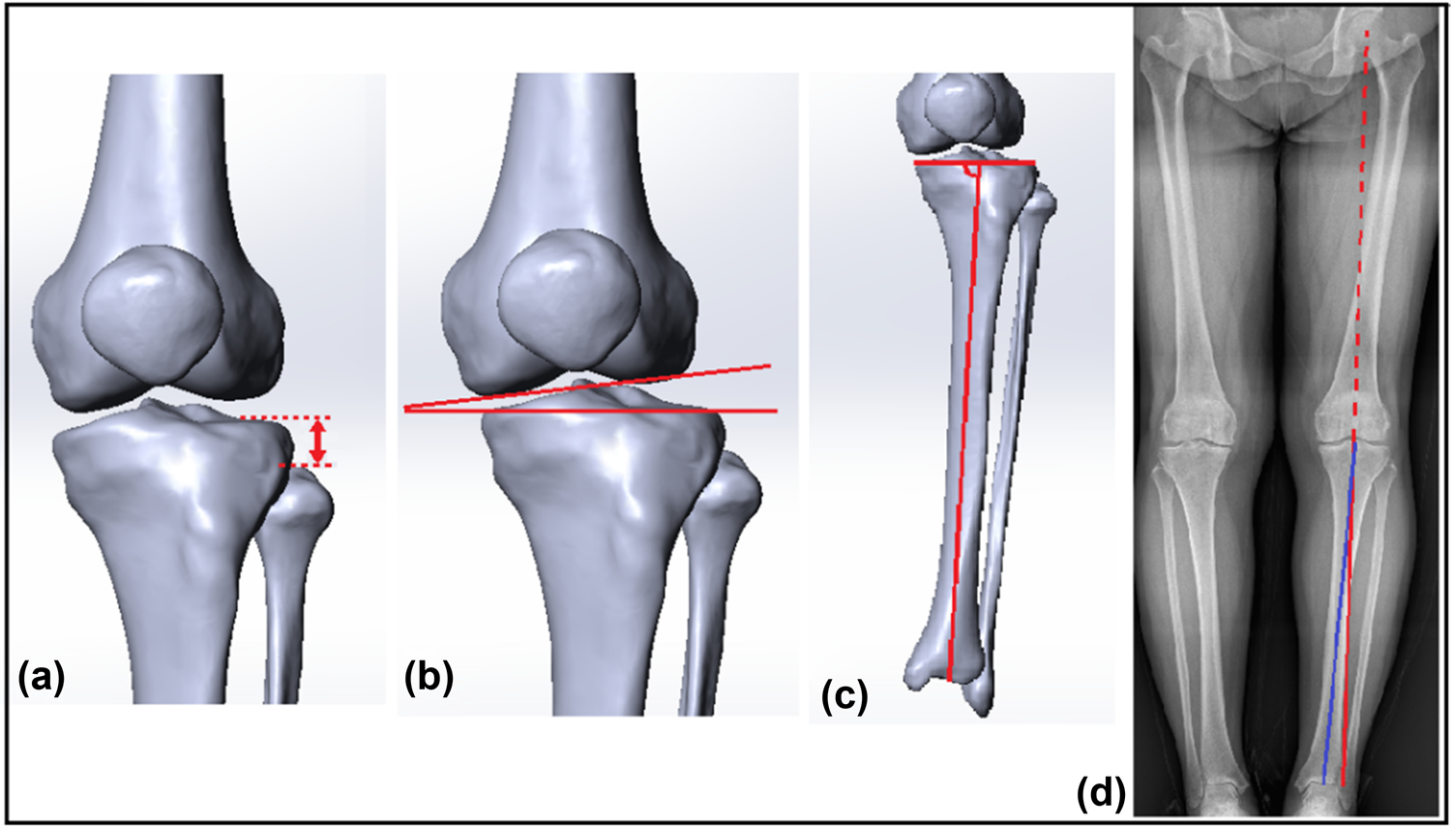
Data measurement. (a) Fibular head height is the distance between the fibular head edge and the horizontal tangent through the lowest point of the lateral tibial plateau. (b) JLCA: the angle between the tangent through the lower edge of the medial and lateral femoral condyles and the tibial plateau plane. (c) MPTA: the medial angle between the tangent through the tibial plateau and the tibial mechanical axis. (d) Hip–knee–ankle angle: the angle between the extension line downward of the femoral mechanical axis (blue solid line) and the tibial mechanical axis (yellow solid line).

Patient CT data were collected and three-dimensional reconstruction of the knee joint was performed by mimics software (version 16.0, Materialise NV, Leuven, Belgium). Fibular head height was defined as the distance between the upper edge of the fibular head and the horizontal tangent passing through the lowest point of the lateral tibial plateau was measured on a three-dimensional model of the knee (Figure 1a). Joint line convergence angle (JLCA) was defined as the angle between the articular surface of the distal femur and the plane of the tibial plateau based on a three-dimensional model of the knee joint (Figure 1b) [9]. Medial proximal tibial angle (MPTA) was defined as the medial angle between the tibial plateau plane and the mechanical axis of the tibia on the full-length anteroposterior X-ray of the standard weight-bearing lower limb (Figure 1c) [10]. Hip–knee–ankle angle was defined as the angle between the extension line from the center of the femoral head to the center of the medial and lateral femoral condyles and the mechanical axis of the tibia on the full-length anteroposterior X-ray of the lower limb in the standard weight-bearing position, and the medial angulation is knee varus (Figure 1d). Kellgren–Lawrence (K–L) grade was as follows: radiographic severity grading criteria for knee osteoarthritis, graded according to the findings of knee degeneration on X-ray: grade 0, no significant abnormal changes on X-ray; grade I, suspected narrowing of the knee joint space or suspected osteophytes; grade II, significant osteophytes, mild narrowing of the joint space; grade III, moderate osteophytes, narrow joint space, mild subchondral osteosclerosis, small range; and grade IV, a large number of osteophytes, severe narrowing of the joint space, significant osteosclerosis, accessible subchondral surface, accompanied by significant joint hypertrophy and deformity [11,12]. All measurements were performed by independent researchers (J.Y. and D.Y.), with a third experienced investigator (J.Y.) making the final decision when differences were significant.

### 2.3 Grouping

The patients were divided into five groups according to the K–L grade of the knee: group A:grade 0, group B:grade I, group C:grade II, group D:grade III and group E:grade IV.

### 2.4 Statistical processing

Statistical analysis was performed using SPSS 25.0 statistical software package (SPSS, USA). Age, height, weight, body mass index, fibular head height, JLCA, MPTA and hip–knee–ankle angle were used as measurement data and conformed to the normal distribution by normal test, expressed as mean ± standard deviation and compared using analysis of variance and pairwise comparison *q* test; gender distribution was expressed as frequency and compared using *χ* ^2^ test. The test level α is equal to 0.05. K–L grade was used as the dependent variable, and age, gender, height, body mass index and fibular head height were included as independent variables in ordinal multivariate logistic regression to analyze the effect of each factor on the occurrence and severity of knee osteoarthritis. Pearson correlation was used to analyze the correlation between fibular head height and hip–knee–ankle angle, and correlation coefficient *r* was used to reflect the degree of correlation between indicators. A receiver operating characteristic (ROC) curve was used for the evaluation of the cutoff point for the fibular head height.

## 3 Results

### 3.1 Comparison of the general data of patients in different groups

A total of 232 patients (232 knees) were finally included in the study, 86 males and 146 females, aged (61.12 ± 10.98) years (range 35–68 years). K–L grade was performed in 28 patients in group A, 31 patients in group B, 49 patients in group C, 53 patients in group D and 71 patients in group E. The differences in age, gender, height, body mass index, fibular head height, JLCA, MPTA and hip–knee–ankle angle among the five groups were statistically significant (*P* < 0.05), and the differences in body weight were not statistically significant (*P* > 0.05, Table 1).

**Table 1:** Comparison of the general data of patients in different groups

Grouping	Cases	Gender (male/female)	Age (years)	Height (cm)	Weight (kg)	BMI (kg/m^2^)
Group A	28	11/17	40.75 ± 5.73	160.07 ± 4.88	60.82 ± 3.76	23.79 ± 1.86
Group B	31	13/18	58.81 ± 5.58*	160.45 ± 7.29	61.64 ± 6.08	24.09 ± 3.38
Group C	49	18/31	61.17 ± 6.43*^#^	161.96 ± 6.48	62.15 ± 5.46	23.82 ± 2.96
Group D	53	20/33	62.17 ± 6.72*^#^	161.57 ± 6.18	62.41 ± 6.47	24.01 ± 2.57
Group E	71	24/47	65.46 ± 7.12*^#▲★^	160.62 ± 6.08	65.47 ± 6.48^*#▲★^	25.45 ± 3.04^*▲★^
Statistics	—	*χ* ^2^ = 0.711	*F* = 304.969	*F* = 0.711	*F* = 5.291	*F* = 3.633
*P*-values	—	0.950	0.000	0.585	0.000	0.007

Note: *: There was significant difference compared with group A. ^#^: There was significant difference compared with group B. ^▲^: There was significant difference compared with group C. ^★^: There was significant difference compared with group D.

### 3.2 Comparison of imaging measurement indicators between different groups

There were significant differences in fibular head height, JLCA, MPTA and hip–knee–ankle angle between different groups (*P* < 0.05). Furthermore, there were significant differences in JLCA and hip–knee–ankle angle (*P* < 0.05), and both JLCA and hip–knee–ankle angle increased with severe aggravation of K–L grade. Furthermore, both fibular head height and MPTA decreased as the K–L grade was severely aggravated. There was no significant difference in fibular head height and MPTA between group B and group C (*P* > 0.05), but there were significant differences in fibular head height and other MPTA (*P* < 0.05; Table 2).

**Table 2:** Comparison of imaging measurement indicators between different groups

Grouping	Cases	Fibular head height (mm)	JLCA (°)	MPTA (°)	Hip–knee–ankle angle (°)
Group A	28	13.34 ± 1.61	0.54 ± 0.09	87.93 ± 1.05	1.86 ± 0.16
Group B	31	11.78 ± 1.34*	0.88 ± 0.16*	86.37 ± 1.67*	3.16 ± 0.324*
Group C	49	11.46 ± 1.27*	1.73 ± 0.12*^#^	86.21 ± 1.49*	4.43 ± 0.67*#
Group D	53	10.07 ± 1.20^*#▲^	3.46 ± 0.34*^#▲^	86.06 ± 1.70*	5.49 ± 1.11^*#▲^
Group E	71	7.46 ± 1.24^*#▲★^	6.49 ± 0.86*^#▲★^	84.46 ± 1.07^*#▲★^	10.33 ± 1.08^*#▲★^
Statistics	—	*F* = 142.173	*F* = 179.437	*F* = 34.432	*F* = 725.932
*P*-values	—	<0.001	<0.001	<0.001	<0.001

Note: *: There was significant difference compared with group A. ^#^: There was significant difference compared with group B. ^▲^: There was significant difference compared with group C. ^★^: There was significant difference compared with group D.

### 3.3 Correlation between fibular head height and knee joint severity and varus deformity

**Figure 2:**
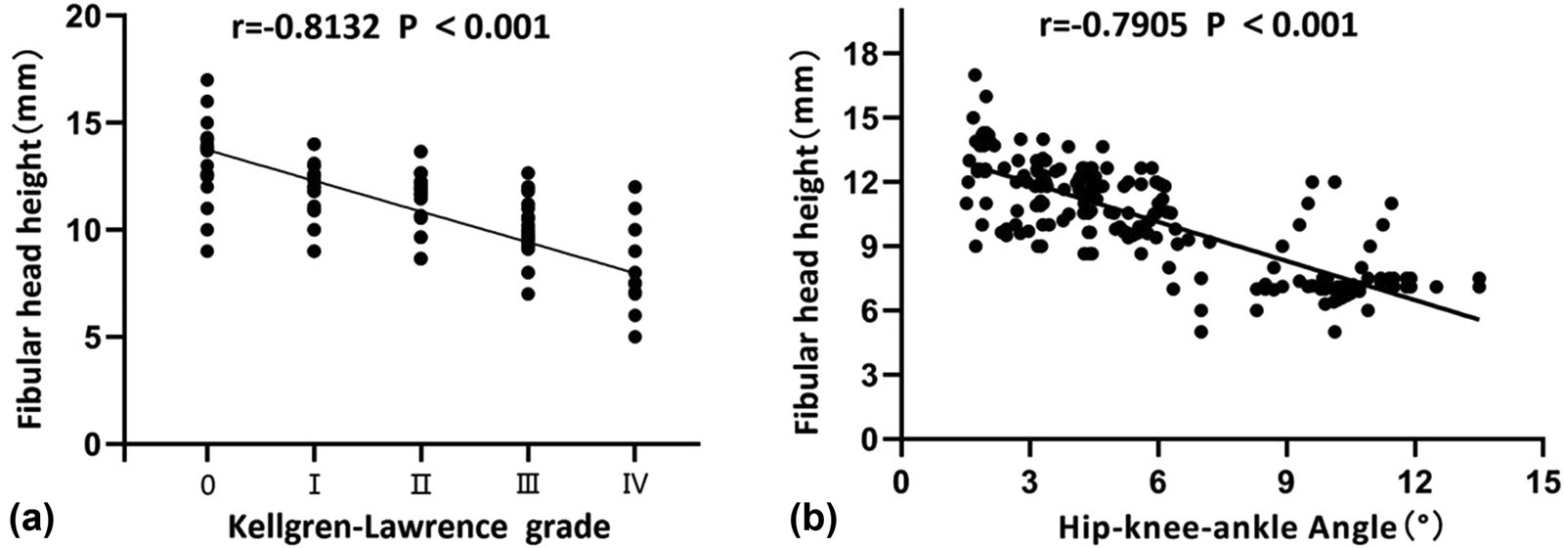
Correlation between fibular head height and knee joint severity (a) and varus deformity (b).

There was a significant negative correlation between K–L grade and fibular head height (*r* = −0.812, *P* < 0.001), that is, the more severe the K–L grade, the lower the fibular head height. Furthermore, there was a significant negative relationship between fibular head height and coxa-knee-ankle angle (*r* = −0.7905, *P* < 0.001), that is, the more severe the grade of knee varus deformity, the lower the fibular head height (Figure 2).

### 3.4 Analysis of influencing factors of K–L grading

The effects of age, gender, height, weight, body mass index and fibular head height on K–L grade of osteoarthritis associated with varus knee deformity were analyzed using ordinal logistic regression that met the proportional odds hypothesis. The results of the parallel line test suggested that the proportional odds hypothesis of the regression model was established and it was suitable to use ordinal logistic regression analysis (*χ* ^2^ = 379.95, *P* = 0.000), the pseudo *R* ^2^ of the model = 0.806, suggesting that the statistical model fitted well. The results showed that fibular head height and body mass index had a significant effect on K–L grade, and their odds ratio (95% confidence interval) were 0.745 (−0.294, −0.862) and 1.246 (−2.781, −0.568), respectively. It is suggested that for every 1 mm increase in fibular head height, the risk of developing osteoarthritis or a 1 grade increase in K–L grade is reduced to 0.745. For each 1 kg/m^2^ increase in body mass index, the risk of developing osteoarthritis or a 1-grade increase in the K–L grade increased by 1.246-fold (Table 3).

**Table 3:** Ordinal logistic regression with K–L grade as the dependent variable

Parameters	*β*	S.E.	Wals	*P*	OR	95% CI
Fibular head height	−1.078	0.110	95.645	0.000	0.745	−1.294, −0.862
Age	0.318	0.038	68.741	0.053	1.134	0.243, 0.394
Height	−0.524	0.174	9.042	0.432	1.045	−0.865, −0.182
Weight	0.672	0.221	9.230	0.534	0.895	0.238, 1.105
BMI	0.325	0.565	8.795	0.019	1.246	−2.781, −0.568
Gender	−0.382	0.316	1.462	0.227	1.023	−1.002,0.237

Note: *β*: regression coefficient, SE: standard error of regression coefficient, OR: odds ratio, CI: confidence interval.

### 3.5 ROC curve analysis

**Figure 3:**
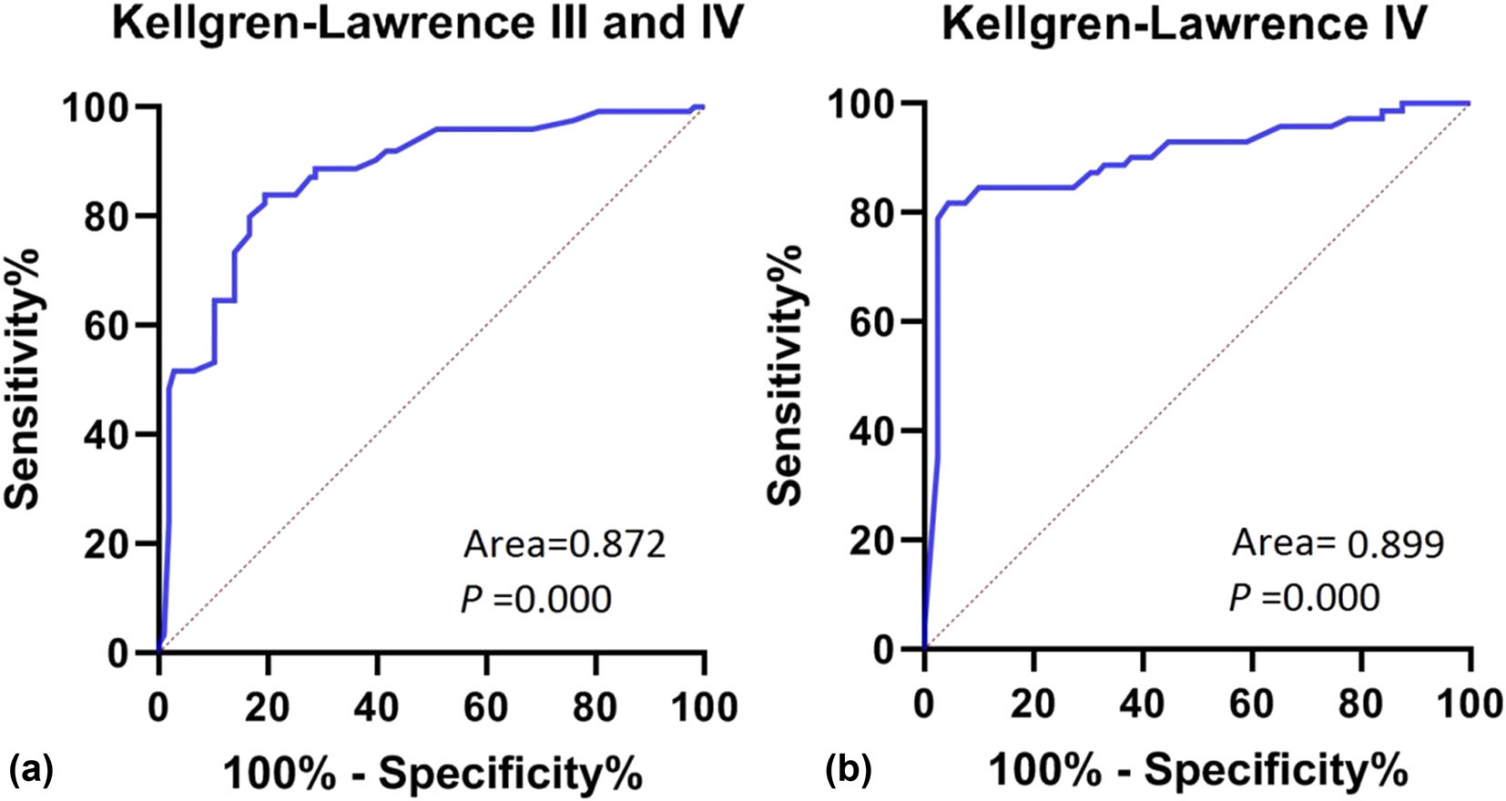
ROC curve analysis, (a) K–L grade III and IV and (b) K–L grade IV.

When K–L grade III and IV knees were considered as disease and normal controls for knees below grade III, ROC curve analysis showed a cutoff value of 10.63 for fibular head height and an AUC of 0.872. In addition, when K–L grade IV knees were considered disease and normal controls of knees below grade IV, ROC curve analysis showed a cutoff value of 8.25 for fibular head height and an AUC of 0.599 (Table 4 and Figure 3).

**Table 4:** ROC curve analysis

Parameters	Disease	Area	*P*	Progressive 95% CI		Cutoff value	Sensibility	Specificity
				Lower limit	Upper limit			
Fibular head height (mm)	(Ⅲ and IV)	0.872	0.000	0.826	0.918	10.63	83.9%	80.6%
Fibular head height (mm)	(IV)	0.899	0.000	0.848	0.915	8.25	81.7%	95.7%

## 4 Discussion

This study innovatively measured image data by three-dimensional reconstruction of the knee and found a correlation between fibular head height and osteoarthritis associated with varus knee deformity. First, the fibular head height of patients with varus knee arthritis was smaller than that of non-osteoarthritis population, and the fibular head height was a risk factor for osteoarthritis in addition to body mass index. Second, the fibular head height was negatively correlated with the severity of osteoarthritis and the degree of varus deformity.

The fibular head is an important ligament and tendon attachment point, including the lateral collateral ligament, arcuate ligament, popliteofibular ligament, biceps femoris muscle and peroneus longus muscle, and the popliteal tendon is indirectly connected to it through the popliteofibular ligament, so its location is closely related to the function of the ligament and tendon [13]. Biomechanical studies have found that compared with the normal population, patients with osteoarthritis associated with varus knee deformity have a higher peak activity level of the peroneus longus muscle and a lower peak activity level of the biceps femoris muscle, while on the first day after PFO, the activity level of the peroneus longus muscle is significantly lower while the peroneus head is shifted downward, and the activity level of the biceps femoris muscle is significantly increased at 6 months after surgery and maintained at a higher level [5]. Theoretically, a decrease in the height of the fibular head will lead to the relaxation and tension reduction of the lateral knee-related ligaments and tendons, and muscles and ligaments are important structures to maintain knee stability [14]. This study found that for every 1-mm increase in fibular head height, the risk of developing osteoarthritis or a 1 grade increase in the K–L grade was reduced to 0.745 and showed that changes in fibular head height in internal turned knee osteoarthritis are not only manifestations of osteoarthritis but also more likely to be a risk factor leading to knee instability, arthritis pathogenesis and progression.

Lateral soft tissues of the knee play an important role in limiting abnormal external rotation of the tibia [15]. The posterolateral knee ligament complex, composed of the lateral collateral ligament, arcuate ligament, popliteofibular ligament and popliteal tendon, produces the greatest limiting force against external tibial rotation when the knee is flexed 30–45° [16]. The maximum limiting force against external tibial rotation at 0–30° of knee flexion is generated by the lateral collateral ligament, the popliteal complex plays a major role in place when the knee continues to flex [17]. This study confirmed that fibular head height was negatively correlated with the severity of osteoarthritis associated with varus knee deformity, that is, the lower the fibular head height, the more severe the severity of knee osteoarthritis. Elevated relative position of the fibular head in patients with inversion osteoarthritis will cause a decrease in lateral soft tissue tension, so instability of the knee in the horizontal position may manifest as abnormal external rotation of the tibia. It has been confirmed that there is indeed an abnormal increase in the external rotation angle of the tibia in patients with osteoarthritis [18,19], and the abnormal external rotation angle of the tibia increases with the severity of arthritis [20]. These studies also confirmed the findings of the present study. Abnormal external rotation of the tibia is an important cause of increased pressure in the medial knee compartment [21,22], while abnormal increase in pressure will exacerbate knee degeneration, such as hyperosteogeny and osteosclerosis [23]. The proximal tibiofibular joint (PTFJ) relieves the stress created by the lateral tibial bending moment. Therefore, the position of the fibular head may affect the function of the PTFJ. There may be a potential correlation between PTFJ inclination (horizontal, oblique) and severity of osteoarthritis associated with varus knee deformity, which requires our further observational studies.

The lateral soft tissues of the knee joint play an important role in maintaining the normal lower limb alignment [24,25,26], and the posterolateral ligamentous complex of the knee joint is the main structure that limits varus deformity of the knee joint. The lateral collateral ligament plays a major role in limiting varus deformity of the knee when the knee is flexed from 0 to 30°, and the biceps femoris plays a major role when flexion exceeds 30° [27]. As the attachment point of the above ligaments and tendons, the relative upward movement of the position of the fibular head may weaken the structural function that limits knee varus, and the worse the stability of the lateral structure of the knee in the coronal plane, the more severe the varus deformity performance. This may be part of the mechanism by which the degree of varus deformity in osteoarthritis associated with varus knee deformity is inversely correlated with fibular head height. Some scholars believe that the force on the fibular head in the vertical direction is a tensile force rather than a compressive force, which may be the mechanical reason for the displacement of the fibular head. However, this study confirmed that in addition to the mechanism by which the fibula affects the pathogenesis and severity of osteoarthritis associated with varus knee deformity, there is a close association with fibular head height.

This study innovatively measured image data by three-dimensional reconstruction of the knee and found a correlation between fibular head height and osteoarthritis associated with varus knee deformity. This study then remains limited. First of all, this study was a single-center study with a limited number of cases and possible selection bias. Second, this study was retrospective and could not describe the dynamic changes in fibular head height and progression of osteoarthritis in each patient. In addition, the effect of fibular head height on knee osteoarthritis still requires mechanical experiments such as finite element for mechanistic studies. Furthermore, this study investigates the correlation between the fibular height and KOA defined according to the radiological K–L classification. However, knee osteophytes and joint space narrowing are better assessed using the OARSI atlas, which may be more beneficial to assess the disease progression of KOA.

In conclusion, the height of fibular head in patients with osteoarthritis associated with varus knee deformity is smaller than that in non-osteoarthritis patients. In addition to body mass index, fibular head height is a risk factor for the pathogenesis of osteoarthritis associated with varus knee deformity; the smaller the fibular head height, the more severe the severity of osteoarthritis and the more severe the degree of varus deformity.
